# Comorbid CAD and ventricular hypertrophy compromise the perfusion of myocardial tissue at subcritical stenosis of epicardial coronaries

**DOI:** 10.1186/s43044-019-0003-5

**Published:** 2019-08-05

**Authors:** Eslam Abbas

**Affiliations:** Kobri El Koba Medical Complex, El Khalifa El Maamoun St. Intersection of El Fangary St, Heliopolis, Cairo 11766 Egypt

**Keywords:** CAD, Ventricular hypertrophy, Structural resistance, Arrhythmia, MI

## Abstract

**Background:**

Most studies of CAD revascularization have been based on and reported according to angiographic criteria which do not consider the relation between the resulting effective flow distal to the stenosis and the demand of a hypertrophied myocardial tissue.

**Results:**

A mathematical model of the myocardial perfusion in comorbid CAD and ventricular hypertrophy, using Poiseuille’s law, indicates that the affected patients are more sensitive to CAD-related hemodynamic changes. They are more prone to develop ischemic complications, mainly non-ST-elevation myocardial infarction (NSTEMI), and arrhythmias than their peers with isolated CAD regarding the same degree of coronary stenosis.

**Conclusion:**

Patients with comorbid CAD and ventricular hypertrophy suffer from myocardial hypoperfusion at subcritical epicardial stenosis. Accordingly, the comorbidity of both diseases should be considered upon designing of the treatment regimen.

## Background

Combined coronary artery disease and ventricular hypertrophy are not uncommon; they both share hypertension, which affects 31% of the world population [1], as a risk factor. Accumulation of atheromatous plaques under tunica intima of the epicardial arteries restricts the blood flow to the supplied cardiac tissue. Chronic high-grade narrowing of the coronary arteries induces subendocardial ischemia during the escalation of the myocardial oxygen demand throughout exercise or stress [2]. The strained myocytes release mediators like adenosine and bradykinin [3, 4], which stimulate vasodilatation and precipitate angina by irritating nerve endings [5].

The treatment strategy for treating CAD aims to improve survival and/or relieve symptoms [6], including dyspnea and stable angina pectoris. This strategy usually involves anti-anginal medications and/or PCI, or CABG in case of complex CAD and/or left main involvement, for achieving those aims. The transition from pharmacotherapy to revascularization is recommended in case of persistence of symptoms and/or the improvement of prognosis [7]. Trials have shown that revascularization by PCI or CABG is more effective than medical therapy alone, in relieving symptoms like angina and dyspnea. Besides, it improves the quality of life by reducing the use of anti-angina drugs and increasing exercise capacity [8–12]. Several studies indicate that PCI, as an initial management strategy in patients with stable coronary artery disease, did not reduce the risk of complications as myocardial infarction or other major cardiovascular events when added to optimal medical therapy [12–15]. However, a recent network meta-analysis study of 100 trials reported improved survival using PCI with new-generation DES compared with initial medical treatment [16].

Generally, PCI and medical therapy should be viewed as complementary, rather than opposing, strategies [17]. Patients with stable coronary artery disease and functionally significant stenoses benefit from the combination therapy of PCI plus optimal medical therapy by showing greater symptomatic improvement [18] and decreasing need for urgent revascularization. However, in patients without ischemia, the outcome appeared to be favorable with optimal medical therapy alone [19].

Significant stenosis has been defined by most studies of CAD revascularization as ≥70% [20] diameter narrowing and/or ≥50% for left main CAD [21, 22]. These criteria have been based on and reported according to an angiographic method. Alternatively, coronary artery stenosis with FFR ≤0.8 is also defined to be significant [23–25]. This criterion has been based on and reported according to an angiophysiological method. The standard values provided by both methods, and so the revascularization decision, do not consider the relation between the resulting effective flow distal to the stenosis and the demand of a comorbid hypertrophied myocardial tissue.

## Model

Hagen-Poiseuille law, which is an analytical solution to the Navier-Stokes equation [26], states that the flow rate *Q* through a coronary vessel is directly proportional to the pressure gradient *∆P* between the aortic root and the right atrium and inversely proportional to the resistance *R* within the vessel, wherein the resistance *R* is inversely proportional to the radius *α* of the vessel elevated to the fourth power and is directly proportional to the blood viscosity *μ* and the vessel length *∆l*. So, by considering a circular cross-section of the vessel:

$$ Q=\Delta  P\frac{\pi {\alpha}^4}{8\mu \Delta  l} $$

when

$$ R=\frac{8\mu \Delta  l}{\pi {\alpha}^4} $$

So:

$$ Q\propto \frac{1}{R}\propto {\alpha}^4\propto \frac{1}{\Delta  l} $$

The blood flow, which is a non-Newtonian fluid, within the circulation does not imitate precisely this law [27], because the equation is applied on a Newtonian fluid in the steady laminar flow moving through a long cylindrical pipe. Still, the law outlines the dominant determinants which influence the blood flow *Q* within the vasculature either in physiological or pathological conditions.

Atherosclerosis commonly affects the epicardial coronary vessels leading to narrowing of the vessel caliber *α*_*e*_ and increase vascular resistance of the supplying vessel *R*_e_, while:

$$ {R}_{\mathrm{e}}\propto \frac{1}{\alpha_e^4} $$

The corresponding supplied myocardial segment does not actually suffer from this blood flow reduction indicated in the above equation. The vasculature of the coronary circulation is arranged in series, in addition to the parallel arrangement, so that the epicardial vascular resistance *R*_e_ is a segmental resistance. The coronary circulation can be divided into two compartments, the large epicardial conduit vessels and the resistance vessels, which are typically less than 300 μm in diameter [28]. Whereas the conduit vessels exert little if any resistance to flow, resistance to flow progressively rises as the vessel diameter of the resistance vessels declines from about 300 μm in the small arteries to less than 100 μm in the arteriolar vessels [29]. Therefore, the total resistance to blood flow *R* comprises mainly the pre-capillary resistance *R*_c_, the resistance of microvasculature *R*_m_, and the negligible resistance of the epicardial or conductance vessels *R*_e_.

$$ R={R}_{\mathrm{c}}+{R}_{\mathrm{m}}+{R}_{\mathrm{e}} $$

Narrowing of the radius of the epicardial vessel, due to atheromatous plaque, will increase the resistance in this vessel, but as:

$$ {R}_{\mathrm{c}}+{R}_{\mathrm{m}}\gg {R}_{\mathrm{e}} $$

the impact of mild to moderate increase of the epicardial resistance *R*_e_ on the overall resistance of the coronary circulation *R* is insignificant.

However, in case of combined coronary artery disease and ventricular hypertrophy, both *R*_e_ and *R*_c_ + *R*_m_ are increased. Microangiogenesis is activated during the pathogenesis of ventricular hypertrophy as a compensatory mechanism to maintain effective blood supply to the hypertrophied tissue. Accordingly, CAD causes an increase in *R*_e_ due to epicardial arterial stenosis, and ventricular hypertrophy increases *R*_c_ + *R*_m_ due to neomicroangiogenesis, i.e., addition of a new microvascular segment.

$$ {R}_{\mathrm{c}+\mathrm{m}}\propto \frac{1}{\alpha_{\mathrm{m}}^4}\propto \Delta  l $$

Consequently, the flow rate *Q*, and so the perfusion of myocardial tissue, diminishes significantly upon subcritical stenosis of the supplying epicardial artery during the pathogenesis of CAD.

As mentioned, the identification of clinically significant stenosis of an epicardial artery depends on an angiographic criterion, its radius *α*_*e*_, and/or an angiophysiologic criterion, the FFR. The graphical representation of the relation between both these criteria of the supplying artery and the perfusion of the supplied myocardial tissue follows a directly proportional relationship represented by a sigmoid-shaped curve, due to the effect of segmental resistance. Myocardial perfusion *ξ* describes the blood flow *Q* in milliliters per minute per cubic centimeter of cardiac muscle volume *V*.

$$ \xi =\frac{Q}{V} $$

**Fig. 1:**
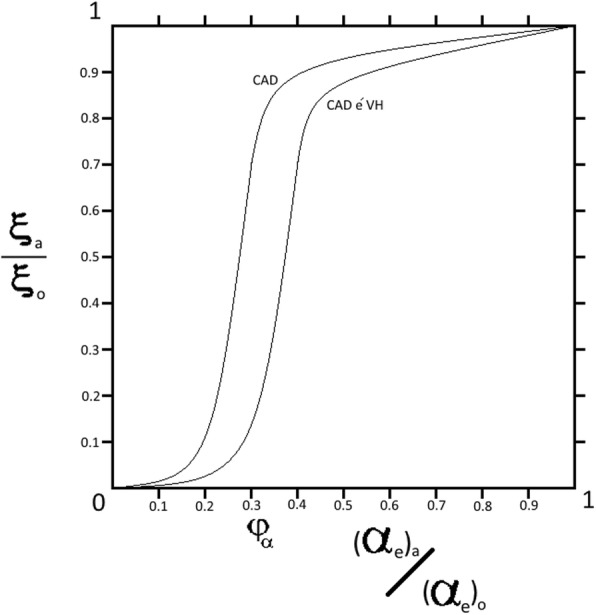
A graphical representation of the relation between the ratio of the perfusion of myocardial tissue supplied by a stenotic epicardial coronary *ξ*_*a*_ to the perfusion in case of hypothetical absence of stenosis *ξ*_0_ and the ratio of the radius of the said stenotic artery (*α*_*e*_)_*a*_ to the radius in case of hypothetical absence of stenosis (*α*_*e*_)_0_. Both ratios are presented by absolute numbers. In isolated CAD, the directly proportional relationship is represented by a sigmoid-shaped curve, wherein the perfusion of myocardial tissue supplied by the said stenotic epicardial coronary *ξ*_*a*_ collapses relatively at a critical stenotic value *ϕ*_*α*_. Comorbid CAD and ventricular hypertrophy shift the curve to the right leading to an increase in the critical stenotic value *ϕ*_*α*_

According to the relation between the radius *α*_*e*_ of an epicardial coronary artery, as an angiographic criterion, and the perfusion *ξ* of the corresponding supplied myocardial tissue represented in Fig. 1, the perfusion *ξ* does not decrease significantly with gradual stenosis in isolated CAD until a critical stenotic value *ϕ*_*α*_ is reached, wherein the perfusion collapses relatively. Clinically, the said critical value *ϕ*_*α*_ is defined as ≥70% radius *α*_*e*_ reduction, significant stenosis [20–22]. However, in patients with comorbid CAD and ventricular hypertrophy, the curve is shifted to the right indicating an increase in the critical stenotic value *ϕ*_*α*_, so that the perfusion *ξ* of the corresponding supplied myocardial tissue collapses relatively at a clinically subsignificant stenosis. The right shift in the said patients depends on the degree of ventricular hypertrophy.

**Fig. 2:**
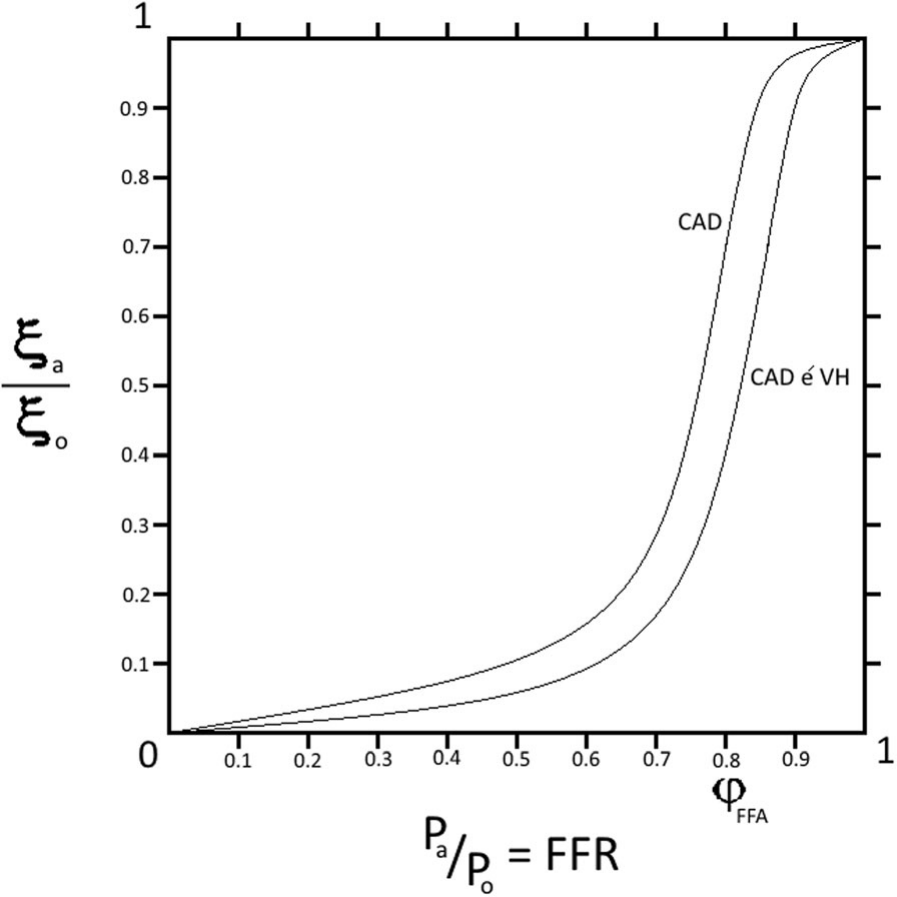
A graphical representation of the relation between the ratio of the perfusion of myocardial tissue supplied by a stenotic epicardial coronary *ξ*_*a*_ to the perfusion in case of hypothetical absence of stenosis *ξ*_0_, and FFR which is the ratio of the pressure distal to the stenosis *P*_*a*_ to the pressure proximal to the stenosis *P*_0_. Both ratios are presented by absolute numbers. In isolated CAD, the directly proportional relationship is represented by a sigmoid-shaped curve, wherein the perfusion of myocardial tissue supplied by the said stenotic epicardial coronary *ξ*_*a*_ collapses relatively at a critical stenotic value *ϕ*_FFR_. Comorbid CAD and ventricular hypertrophy shift the curve to the right leading to an increase in the critical stenotic value *ϕ*_FFR_

Additionally, the relation between another angiophysiologic criterion; the fractional flow reserve FFR, within a stenotic epicardial artery; and the perfusion *ξ* of the corresponding supplied myocardial tissue, as represented in Fig. 2, indicates that the perfusion *ξ* is not meaningfully reduced with the gradual decrease of FFR until a critical value *ϕ*_FFR_ is reached, wherein the perfusion *ξ* collapses relatively. Clinical trials defined the said critical value *ϕ*_*FFR*_ as an FFR ≤0.8 [23–25, 30, 31]. However, in patients with combined CAD and ventricular hypertrophy, the curve shows a right shift, which is directly proportional to the degree of ventricular hypertrophy, indicating an increase in the critical stenotic value *ϕ*_FFR_, so that the perfusion *ξ* of the corresponding supplied myocardial tissue collapses relatively at a clinically subsignificant reduction in the FFR.

The proposed model gives a more sensitive formula to detect the critical stenosis, which takes into account the demand of the supplied bulky myocardium. The isolated CAD curve is a logistic function, wherein *x* represents the critical stenosis and *k* is the curve slope:

$$ f(x)=\frac{1}{1+{e}^{- kx}} $$

In patients with comorbid CAD and ventricular hypertrophy, the curve is shifted to the right by *a* yielding *x*` as a representation of the critical stenosis:

$$ f(x)=\frac{1}{1+{e}^{-k\left(x`-a\right)}} $$

then

$$ x`=x+a $$

wherein the curve shift *a* is directly proportional to the difference in muscle bulk *∆M* which is obtained by echocardiogram and wherein the muscle bulk is considered hypertrophied when LVMI >115 g/m^2^ in males and LVMI >95 g/m^2^ in females [32]:

$$ a\propto \Delta  M $$

$$ a=\omega \Delta  M $$

where the value of the constant *ω* can be obtained experimentally. So, the percentage of the critical patency in patients with comorbid CAD and ventricular hypertrophy *x*` is:

$$ x`=x+\left(\omega \Delta  M\right) $$

## Results

*Individuals with pathological ventricular hypertrophy are more sensitive to hemodynamic changes of the coronary circulation or pathologies that reduce the coronary reserve.* Ventricular hypertrophy stresses the subendocardial myotissue due to increasing the structural resistance of the coronary circulation. The said stress is ameliorated by compensatory functional changes to sustain the normal coronary blood flow. Although during vigorous exercise, the compensatory capability of the coronary flow reserve is exhausted under the effect of demand upsurge and shortened diastolic period. Occasional hemodynamic disturbances or subclinical pathologies, which lessen the maximum coronary reserve, may lead to selective subendocardial hypoperfusion.

*Comorbid CAD and ventricular hypertrophy cause the subendocardial tissue to suffer, during exercise or stress, from ischemia at an angiographically subsignificant stenosis in the supplying epicardial artery.* CAD primes the structural resistance of the neomicrovasculature of the hypertrophied tissue. So subcritical stenosis of the corresponding epicardial artery, mainly due to atherosclerosis, causes the total resistance to rise effectively to reduce the flow rate and exhaust the reactive compensatory mechanisms. The curve shift to the right in the said patients does not affect the risk of myocardial infarction, *yet they are more susceptible to and usually presented by non-ST-elevation myocardial infarction (NSTEMI), with higher rates of transition from ischemia to necrosis in the affected hypertrophied endocardial tissue.* Increased muscle bulk shifts the endocardium away from the main blood supply. Besides, subjection to higher extravascular pressure depletes the functional vasodilator reserve in long-standing pathological hypertrophy.

*Patients with combined CAD and ventricular hypertrophy have a higher risk to develop arrhythmias than their peers who suffer from isolated CAD.* In pathological hypertrophy, the neomicroangiogenesis shows anatomical and architectural dysgenesis [33] in relation to the hypertrophied tissue [34]. The said dysgenesis leads to failure of the coronary bed to uniformly supply the cardiac muscle, rendering foci within the hypertrophied muscle bulk at greater risk of ischemic injury. These stressed foci can be arrhythmogenic upon increased cardiac demand leading to serious arrhythmia and sudden cardiac death.

## Discussion

During cardiac catheterization, the main determinants of revascularization therapy in CAD patients are either angiographic or angiophysiological criteria to identify the clinically significant stenosis. The said determinants depend on the relation between the size of the insinuated plaque and the vascular diameter. A stenosis, which reduces the radius of the epicardial vessel by 70% or the fraction flow reserve value ≤0.8, is considered significant. These standard values, provided by both methods to identify a stenotic lesion as significant, do not consider the relation between the resulting effective flow distal to the stenosis and the demand of a hypertrophied myocardial tissue.

Pathological cardiac hypertrophy is a condition that is characterized by the thickening of the heart muscle, a decrease in the size of the chambers of the heart, and a reduced capacity of the heart to pump blood to the tissues and organs around the body. It is associated with contractile dysfunction, interstitial fibrosis, and re-expression of fetal cardiac genes, such as genes coding natriuretic peptides and the β-myosin heavy chain [35, 36]. Two common causes of pathological cardiac hypertrophy are hypertension and heart valve stenosis [37]. On the other hand, physiological cardiac hypertrophy can be provoked by exercise training [38] and can lead to an increased cardiac size that is characterized by normal cardiac morphology with an enhanced cardiac function [39]. Although both types of cardiac hypertrophy are initiated by an overload to the heart, the distinct differences between the two can be attributed to the type of overloading stimuli. Yet, the reason why some cardiac overloading stimuli are beneficial while others are deleterious is unclear [40].

The basic pathogenesis of ventricular hypertrophy implicates a multifaceted process that demonstrates a high degree of cellular and molecular intricacy across multiple signaling pathways [41]. Angiogenesis is triggered during this process by increased cardiac work and oxygen demand, in an attempt to normalize maximal myocardial perfusion and capillary domains to sustain oxygen delivery. A limitation of capillary growth will increase diffusion distance for oxygen, while inadequate arteriolar growth will reduce maximal tissue perfusion. Pathogenesis of hypertrophy is categorized into pressure overload-induced, volume overload-induced, thyroxin-induced, and exercise-induced models according to the stimulus for increasing muscle bulk. In exercise-induced and thyroxin-induced models, angiogenesis and arteriogenesis are well documented experimentally [42]. While in other models, there is a considerable variation in the reports of the literature about the extent and pattern of angiogenesis and the consequential coronary microvascular resistance. The reasons for the discrepancy between these studies are not evident, but the duration of the hypertrophy and the specificity of the stimulus may play a role.

Mathematically, angiogenesis increases the coronary microvascular *structural* resistance, due to the addition of a new microvascular segment. However, in vivo, structural resistance can be modulated by *functional* changes, wherein autoregulatory adjustments involving the vasodilator reserve may ameliorate the said structural resistance escalation. Well-trained athletes with physiological cardiac hypertrophy show a proportional increase of cardiac myocytes and coronary vasculature with no change in the proportion of extracellular collagen [43]. These structural modulations are accompanied by functional adaptations resulting in a compensatory exponential coronary reserve and vasodilator capacity. Functional adaptations can include changes in neurohumoral control and changes in local vascular control mechanisms [44, 45]. In pathological hypertrophy, pathological features of the strained neoangiogenesis halt the functional compensation for the structural increase in the microvascular resistance. Endothelium-dependent vasodilation is markedly impaired in the coronary microvessels of patients with hypertension-induced ventricular hypertrophy [46–48]. Accordingly, severe ventricular hypertrophy is associated with a reduction in coronary vascular reserve [49–51].

Myocardial infarction is mainly caused by rupture of vulnerable fibroatheromatous plaque forming a thrombus that interferes with myocardial blood supply leading to excessive ischemia and then necrosis [52]. Usually, soft non-stenotic plaques are more susceptible to rupture, causing major cardiovascular events [53]. The vulnerability of the plaque depends on lesion-specific characteristics like thin fibrous cap, large lipid-rich necrotic core, increased plaque inflammation, positive vascular remodeling, increased vasa-vasorum neovascularization, and intra-plaque hemorrhage [54]. Therefore, the comorbidity between CAD and ventricular hypertrophy does not affect the risk of developing MI. However, patients with the said comorbid diseases have higher rates of transition from ischemia to necrosis in the affected endocardial tissue, due to increase diffusion distance for oxygen and exhaustion of the functional compensation. They are also more susceptible to and usually presented by NSTEMI, due to the sensitivity of the endocardial myotissue to the equilibrium between the structural resistance of microvasculature and the reactive functional modulation.

Myocardial ischemia is characterized by ionic and biochemical alterations, creating an unstable electrical substrate capable of initiating and sustaining arrhythmias [55]. Theoretically, onerous angiogenesis in pathological hypertrophy shows patterns of anatomical and architectural dysgenesis rendering foci within the hypertrophied muscle bulk at greater risk of ischemic injury. The said stressed foci acquire different electrochemical properties, due to defective function of ATPase-dependent pumps, leading to tissue heterogeneity. Theses foci become arrhythmogenic, especially with increased cardiac demand during above-normal exercise or severe stressful conditions, leading to functional re-entry. Hence, the presence of ventricular hypertrophy is associated with a greater risk of sustained arrhythmias [56].

## Conclusion

The mathematical model establishes that ventricular hypertrophy increases the vascular structural resistance of the coronary circulation due to neomicroangiogenesis. So, patients with comorbid CAD and ventricular hypertrophy suffer, due to exhaustion of functional compensation, from complications of myocardial hypoperfusion at angiographically subsignificant coronary artery stenosis. Accordingly, these patients are more susceptible to NSTEMI, serious arrhythmias, and sudden cardiac death than patients with isolated CAD. Upon confirmation of such results by large investigational studies, the said results should be taken into account during designing the treatment strategy of the said patients.

## Data Availability

All datasets, on which the conclusions of the manuscript rely, are presented in the main paper.
